# Here We Go Again: Age Differences in Adapting Emotion Regulation Strategies Across Repeated Stressors

**DOI:** 10.1093/geroni/igaf122.638

**Published:** 2025-12-31

**Authors:** Gloria Luong

**Affiliations:** Colorado State University, Fort Collins, Colorado, United States

## Abstract

After decades of research on emotion regulation and aging, questions remain regarding for whom and under which conditions we observe age-related advantages or disadvantages in emotion regulation in response to stressors. The current research investigated the degree to which younger and older adults adapt their emotion regulation strategies across repeated exposures to a standardized psychosocial laboratory stressor, the Trier Social Stress Test (TSST). In the Health and Daily Experiences (HEADE) study, 244 participants (138 younger adults, 106 older adults) completed 3 administrations of the TSST across three different days over one week. Participants reported their positive and negative affect at baseline and immediately after the task, as assessments of their affect reactivity to the stressor. They also rated the degree to which they used a variety of emotion regulation strategies during the task. Results revealed that although at the first lab session we found no age differences in positive or negative affect reactivity, these age differences widened with repeated stressor exposures such that older adults showed elevated affect reactivity over younger adults. Moreover, we found that although cognitive reappraisal was an effective strategy for reducing positive and negative affect reactivity and older adults used this strategy more frequently than younger adults at the second and third lab sessions but they still showed elevated affect reactivity. These findings suggest that even though older adults may use more effective strategies more frequently than younger adults, they may do so in an effort to downregulate their high levels of affect reactivity.

